# A Large-Scale Pattern of Ontogenetic Shape Change in Ray-Finned Fishes

**DOI:** 10.1371/journal.pone.0150841

**Published:** 2016-03-04

**Authors:** Hilary R. Katz, Melina E. Hale

**Affiliations:** 1 Graduate Program in Integrative Biology, University of Chicago, Chicago, IL, 60637, United States of America; 2 Department of Organismal Biology and Anatomy, University of Chicago, Chicago, IL, 60637, United States of America; INRA, FRANCE

## Abstract

Fishes exhibit a remarkable diversity of body shape as adults; however, it is unknown whether this diversity is reflected in larval stage morphology. Here we investigate the relationship between larval and adult body shape as expressed by body elongation. We surveyed a broad range of ray-finned fish species and compared body shape at larval and adult stages. Analysis shows that the vast majority of fish are more elongate at the larval stage than at the adult stage, and that adults display greater interspecies variation than larvae. We found that the superorder Elompomorpha is unique because many species within the group do not follow the observed elongation trends. These results indicate that much of the diversity observed in adults is achieved in post-larval stages. We suggest that larval morphology is subject to common constraints across the phylogeny.

## Introduction

Comparing and categorizing species at their adult stage has shown that ray-finned fish species exhibit a wide diversity of body shapes [1–3]. Studies investigating shape diversity at the larval stage indicate that there is less diversity in larvae than adults [4]. However, such work at the larval stage has been limited to a handful of species in one environment or species within a single family [4–5]. In this study, we examine larval and adult body shape diversity on a broader scale. By surveying a wide range of species, we can investigate trends across the phylogeny. We can also identify outlier groups that may be of use as case studies for finding developmental mechanisms for shape diversification.

A particularly informative measure of shape is body elongation [1–3] because it captures much of the axial diversity in adults [2]. The diversity of elongation is associated with a variety of factors including development, locomotion, and physiology. Early developmental processes of axial patterning may constrain larval body shape. Axial morphology could also have evolved to support particular locomotor strategies [6] or to accommodate physiological and behavioral requirements such as respiration [7], burrowing [8], and feeding [9].

Understanding larval body shape, its comparison to adult shape and trends across fishes, are fundamental to understanding fish biodiversity as well as development and evolution of body shape. Here we set out to answer the following questions: Is the diversity in elongation observed in adult fish reflected in the early larval stage? Is there a difference in the variability of body elongation between larval and adult stages? And are there different developmental trajectories by which the adult elongation state is achieved?

## Methods

We collected images from at least one species of fish from every order of the subclass Actinopteri. Orders and families were based on Eschmeyer’s catalogue of fishes [10]. Images were collected from online databases (i.e. FishBase) as well as the literature [S1 Table and S2 Table]. By far our largest source of images of larvae was Jones et al. (1978) [11]. Species from Jones et al. (1978) were selected using random sampling within families while others were selected based on availability to fill in gaps. Images were used from larvae that were as close to hatching as possible so that the larvae measured fell within the same life history stage. At least one image of a larval fish and one of an adult fish were collected for each species. In many clades, images of larvae are rare, and to be consistent across the sample, we selected one individual per species for use in analysis. If we were able to acquire multiple measurements for a species, we used three criteria to objectively decide which one to include. First, an image with a scale was taken over one without. Because we were measuring a dimensionless number, a scale was not necessary for our analysis. Second, many of the images of larvae are only available as drawings or traces, particularly in the older literature. However, if a photo was available, we selected it over a drawing. Finally, an image from a primary publication was selected over other sources. It should be noted, however, that results did not change when data were analyzed using averages of data from multiple images.

**Fig 1:**
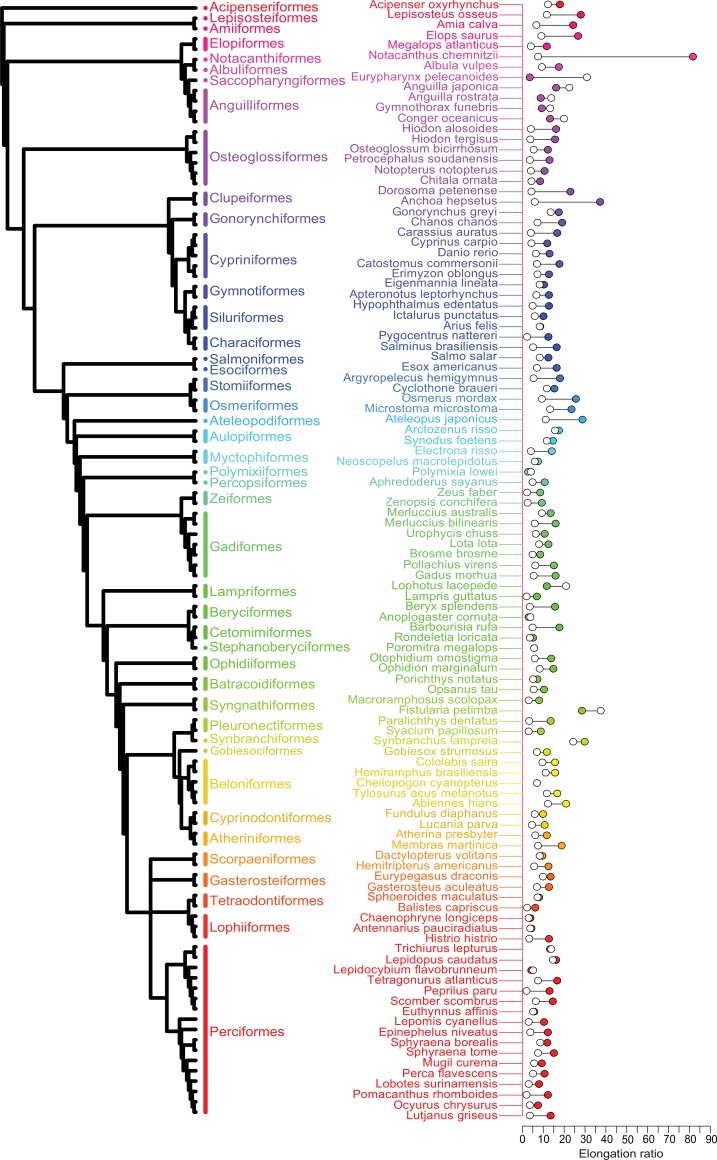
Individual larval and adult elongation ratios. A phylogeny of all species utilized in this study assembled for visualization purposes: orders are indicated in different colors with arbitrary branch lengths. Genus and species name for every species measured in this study with corresponding larval (filled circle) and adult (open circle) elongation ratios (see S1 Table for exact values). The measurements plotted are the same as those used for statistical analyses (see methods section for selection criteria). Colors correspond to the orders from which the species were selected.

Elongation ratio was calculated as a ratio of length to depth and is dimensionless. Length was measured from the center of eye to end of the caudal fin so that elongate snouts would not be included in the measurement. Previous studies have utilized a number of different strategies for measuring depth [2,12]. Instead of measuring the maximum depth, we wanted to measure at a body landmark that could be readily identified and comparable in both larvae and adults. For this reason, we measured depth at the anus, a method previously implemented by Parichy et al. (2009) [12]. Depth did not include the fin fold in larvae or any of the median fins in adults. We did not include these structures in our measurements because our goal was to measure comparable structures at the larval and adult stages. Measurements were taken in ImageJ [13], and statistical analysis was completed in R [14]. We also assembled a phylogeny of all species utilized in this study for visualization purposes (Fig 1). The chimeric phylogeny was assembled, at the order level, primarily from Near et al. (2012) [15]. At the species level, we incorporated several different phylogenies [15–22].

There were two extreme outliers in our data set: *Notacanthus chemnitzii* (snubnosed spiny eel) and *Eurypharynx pelecanoides* (pelican eel). *N*. *chemnitzii* had the greatest larval elongation ratio and was more than twice as elongate as the second most elongate specimen. *E*. *pelecanoides* had a very elongate adult form and an extremely low elongation ratio as a larva. We performed statistical analyses without these two species because we are interested in general group trends and these two species have very distinct characteristics. The results without these animals are presented below, but overall findings are consistent with or without the inclusion of these values.

## Results and Discussion

We first investigated differences between life history stages by comparing elongation ratio between the larval and adult data of each species (Fig 1). Most species achieve their final adult morphology by becoming less elongate than their larval form (96 out of the 108 species sampled). The other 12 species became more elongate through ontogeny. Of these 12 species, five were anguilliformes, or true eels, while the other seven were scattered throughout the phylogeny. Of the 108 species, five showed less than a 10% change in elongation ratio. These five species were scattered across the phylogeny (*Euthynnus affinis*, *Trichiurus lepturus*, *Cheilopogon cyanopterus*, *Poromitra megalops*, and *Arius felis*).

**Fig 2:**
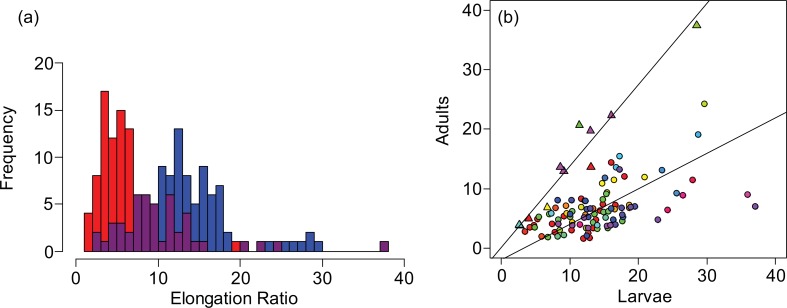
Comparison of larval and adult elongation ratios. (a) The distribution of larval (blue) and adult (red) elongation ratios. The number of species (x-axis) observed for a given elongation ratio (y-axis). (b) Larval elongation ratio plotted against adult elongation ratio. Each point represents a single species and colors correspond to the orders as presented in Fig 1. The regression line is plotted for species that demonstrated a decrease in elongation ratio through ontogeny (circles) as well as for those species that demonstrated an increase in elongation ratio (triangles). Colors correspond to the orders as presented in Fig 1.

We compared the average and distribution of elongation ratios of larval and adult stages, to determine whether the body shape diversity observed in adults is reflected in the larval stage. Larvae (mean±SE, 13.3±0.591) had a significantly greater mean elongation ratio than the adults (7.43±0.551) (ANOVA, p<0.001, Fig 2A), which is consistent with the decrease in elongation ratio observed in most of the species (Fig 1). The adults and larvae also had significantly different distributions of elongation ratios (Komogrov Smirnov test p<0.001; Fig 2A) with a greater coefficient of variation in adults (76.7%) than in larvae (46.1%). Our survey shows that this greater elongation diversity in adults is a broad trend across the fish phylogeny, and not limited to the handful of species that have been previously investigated [4]. Because there is more diversity in elongation in adults than larval counterparts, these data suggest that much of the diversity observed in adults is achieved through post-larval development. This adds a new perspective to previous investigations of elongation in fishes, which propose that diversity is produced by changes in the number or size of somites that are established at the embryonic stage [1, 3].

Differences between larval and adult elongation diversity may be influenced by factors acting at different developmental stages. The evolution of body shape may be influenced by locomotor demands. Larval fish experience primarily intermediate Reynolds numbers (20 < Re < 1000) [23–24], where undulation is a particularly effective form of locomotion [23]. This form of locomotion benefits from a more elongate shape that can propagate waves along the entire body. As body size and swimming speed increase through ontogeny, fish spend more time at high Re (Re >> 1000) [23–24], where they may utilize a broader range of propulsive strategies efficiently. Body shape may also be influenced by physiological factors such as respiration. Larval fish absorb oxygen and ions through the skin, which may constrain post-cranial morphology [7].

To test for association in elongation ratio between larval and adult stages, we performed a reduced major axis regression between larval and adult stages (Fig 2B). For species that showed a decrease in elongation through ontogeny, larval elongation ratio was weakly correlated with adult elongation ratio (R^2^ = 0.322, slope = 0.339, intercept = 1.70, p<0.0001), suggesting that the adult elongation ratio may be independent of the initial larval elongation ratio. However, there was a strong correlation between the two stages for the species that showed the reverse trend (R^2^ = 0.943, slope = 1.33, intercept = 0.516, p<0.0001). Among species that show the reverse trend, the high R^2^ value indicates that adult elongation ratio is highly predictable from larval elongation. Because these species occur across the phylogeny, the same developmental strategy may have evolved multiple times in species that become more elongate through ontogeny.

This study intended to investigate broad trends in elongation, but there are certainly variations within smaller groups that could be investigated further. For example, the superorder Elopomorpha was uniquely variable among the taxa we examined. All anguilliform, or true eel, species sampled showed an increase in elongation ratio through ontogeny. *Notacanthus chemitzii* (snubnosed spiny eel) and *Eurypharynx pelecanoides* (pelican eel) were both major outliers. In contrast, *Megalops atlanticus* (atlantic tarpon) and *Elops saurus* (ladyfish) showed the more common decrease in elongation ratio. Because of this remarkable morphological range in both larvae and adults, we suggest that Elopomorpha would be a valuable group in which to examine the development of body shape and elongation. Since this group includes fewer species and has a well-supported origin [15], phylogenetic comparative methods could be applied to tease out the phylogenetic signal in the relationship between larval and adult body shape.

## Supporting Information

S1 Table Primary values and sources. (left to right) Species, larval and adult elongation ratio values, larval image source, and adult image source for every specimen analyzed in this study. These raw elongation values were used for all statistical tests described in this study. MAB stands for Jones et al., 1987 [11].
(DOCX)

S2 Table Secondary values and sources. These values were not included in the statistical analyses since we were not able to find a second source for every study. We chose to include these sources for the benefit of individuals looking to find multiple online sources for larval and adult stages.
(DOCX)

## References

[1] Ward AB, Brainerd EL. Evolution of axial patterning in elongate fishes. Biol J Linnean Soc. 2007; 90: 97–116. 10.1111/j.1095-8312.2007.00714.x

[2] Claverie T, Wainright P. A morphospace for reef fishes: Elongation is the dominant axis of body shape evolution. PLOS ONE. 2014; 9(11): e1128732 10.1371/journal.pone.0112732 PMC4237352 25409027

[3] Ward AB, Mehta RS. Axial elongation in fishes: using morphological approaches to elucidate developmental mechanisms in studying body shape. Integr Comp Biol. 2010; 50(6): 1106–1119. 10.1093/icd/icq029 21558262

[4] Strauss RE, Fuiman LA. Quantitative comparisons of body form and allometry in larval and adult Pacific sculpins (Teleostei: Cottidae). Can J Zoology. 1985; 63(7): 1582–1589. 10.1139/z85-234

[5] Fuiman LA. Descriptions and comparisons of catostomid fish larvae: northern Atlantic drainage species. T Am Fish Soc. 1979; 108(6): 560–603. 10.1577/1548-8659(1979)108<560:DACOCF>2.0.CO;2

[6] Webb PW. Form and function in fish swimming. Sci Am. 1984; 251: 72–82.

[7] Hale ME. Developmental change in the function of movement systems: transition of the pectoral fins between respiratory and locomotor roles in zebrafish. Integr Comp Biol. 2014; 54(2): 238–249. 10.1093/icb/icu014 24748600 PMC4097112

[8] Herrel A, Choi HF, Dumont E, De Schepper N, Vanhooydonck B, Aerts P, et al Burrowing and subsurface locomotion in anguilliform fish: behavioral specializations and mechanical constraints. J Exp Biol. 2011; 214(8): 1379–1385. 10.1242/jeb.051185 21430215

[9] Toline CA, Baker AJ. Foraging tactic as a potential selection pressure influencing geographic differences in body shape among populations of dace (Phoxinus eos). Can J Zoolog. 1993; 71(11): 2178–2184. 10.1139/z93-306

[10] Eschmeyer W, Fong D. Species by family/ subfamily. Catalog of Fishes. 2015; electronic version (accessed 1 January 2015).

[11] Jones PW, Martin FD, Hardy JD, Johnson GD, Fritzsche RA, Drewry GE. Development of fishes of the Mid-Atlantic Bight. An atlas of egg, larval and juvenile stages. Fish and Wildlife Service, U.S. Department of the Interior; 1978.

[12] Parichy DM, Elizondo MR, Mills MG, Gordon TN, Engeszer RE. Normal table of postembryonic zebrafish development: staging by externally visible anatomy of the living fish. Dev Dynam. 2009; 238(12): 2975–3015. 10.1002/dvdy.22113 PMC3030279 19891001

[13] Schneider CA, Rasband WS, Eliceiri KW. NIH to ImageJ: 25 years of image analysis. Nat. Methods. 2012; 9: 671–675. 22930834 10.1038/nmeth.2089 PMC5554542

[14] R Core Team. R: a language and environment for statistical computing. R foundation for Statistical Computing, Vienna, Austria; 2013.

[15] Near TJ, Eytan RI, Dornburg A, Kuhn KL, Moore JA, Davis MP, et al Resolution of ray-finned fish phylogeny and timing of diversification. PNAS. 2012; 109(34): 1398–13703. 10.1073/pnas.1206625109 PMC3427055 22869754

[16] Near TJ, Dornburg A, Eytan RI, Keck BP, Smith WL, Kuhn KL, et al Phylogeny and tempo of diversification in the superradiation of spiny-rayed fishes. PNAS. 2013; 110(31): 12738–12743. 10.1073/pnas.1304661110 23858462 PMC3732986

[17] Yoon M, Kim KY, Bang IC, Nam YK, Kim DS. Complete mitogenome sequence of the Chinese medaka *Oryzias sinensis* (Teleostei: Beloniformes) and its phylogenetic analysis. Genes Genom. 2011; 33: 307–312. 10.1007/s13258-010-0154-y

[18] Miya M, Friedman M, Satoh TP, Takeshima H, Sado T, Iwasaki W, et al Evolutionary origin of the Scombridae (Tunas and Mackerels): members of a paluogene adaptive radiation with 14 other pelagic fish families. PLOS ONE. 2013; 8(9): e73535 10.1371/journal.pone.0073535 24023883 PMC3762723

[19] Sullivan JP, Lundberg JG, Hardman M. A phylogenetic analysis of the major groups of catfishes (Teleostei: Siluriformes) using *rag1* and *rag2* nuclear gene sequences. Mol. Phylogenet. Evol. 2006; 41: 636–662. 10.1016/j.ympev.2006.05.044 16876440

[20] Inoue JG, Miya M, Miller MJ, Sado T, Hanel R, Hatooka K, et al Deep-ocean origin of the freshwater eels. Biol. Lett. 2010; 6: 363–366. 10.1098/rsbl.2009.0989 20053660 PMC2880065

[21] Wang X, Gan X, Li J, Mayden RL, He S. Cyprinid phylogeny based on Bayesian and maximum likelihood analysis of partitioned data: implications for Cyprinidae systematics. Sci China Life Sci. 2012; 55(9): 761–773. 10.1007/s11427-012-4366-z 23015124

[22] Lavoué S, Sullivan JP. Simultaneous analysis of five molecular markers provides a well-supported phylogenetic hypothesis for the living bony-tongue fishes (Osteoglossomorpha: Teleostei). Mol Phylogenet Evol. 2004; 33(1): 171–185. 10.1016/j.ympev.2004.04.021 15324846

[23] Müller UK, van Leeuwen JL. Swimming of larval zebrafish: ontogeny of body waves and implications for locomotory development. J Exp Biol. 2004; 207(5): 853–868. 10.1242/jeb.00821 14747416

[24] Webb PW, Weihs D. Functional locomotor morphology of early life history stages of fishes. T Am Fish Soc. 1986; 115(1): 115–127. 10.1577/1548-8659(1986)115<115:FLMOEL>2.0.CO;2

