# Supplementary material for: A Large-Scale Pattern of Ontogenetic Shape Change in Ray-Finned Fishes
Source: PLoS One. 2016 Mar 4;11(3):e0150841. doi: 10.1371/journal.pone.0150841 (PMC4778928; doi:10.1371/journal.pone.0150841)
Supplement: S1 Table — (left to right) Species, larval and adult elongation ratio values, larval image source, and adult image source for every specimen analyzed in this study. These raw elongation values were used for all statistical tests described in this study. MAB stands for Jones et al., 1987 [11]. (DOCX) [file pone.0150841.s001.docx]

**S1 Table: Primary values and sources.** (left to right) Species, larval and adult elongation ratio values, larval image source, and adult image source for every specimen analyzed in this study. These raw elongation values were used for all statistical tests described in this study. MAB stands for Jones et al., 1987 [11].

| Species | larval,  adult elongation ratio | Larval source | Adult Source |
| --- | --- | --- | --- |
| *Acipenser oxyrhyncus* | 17.97,  12.07 | O'Connor JM, Alber JB, Arvidson LG. 1981 Development and identification of larval Atlantic sturgeon (*Acipenser oxyrhynchus*) and shortnose sturgeon (*A. brevirostrum*) from the Hudson River estuary, New York. *Copeia*. 711-717. | MAB I: Goode GB, and a Staff of Associates. 1884 |
| *Lepisosteus osseus* | 27.93,  11.53 | MAB I: Mansueti AJ, Hardy Jr. JD, after Kerr JG. 1919 | MAB I: Suttkus RD. 1963 |
| *Amia calva* | 24.21,  6.50 | MAB I: Dean B. 1896 | MAB I: Goode GB, and a Staff of Associates. 1884 |
| *Elops saurus* | 26.46,  8.96 | MAB I: Gehringer JW. 1959a | MAB I: Goode GB, and a Staff of Associates.1884 |
| *Megalops atlanticus* | 11.48,  4.00 | Fahay MP. 2007 *Early stages of fishes in the Western North Atlantic Ocean*. NAFO. From Smith. 1980 | fishbase: Meatl_u1.jpg by Garcia CB. |
| *Notacanthus chemnitzii* | 81.74,  7.29 | Fahay MP. 2007 *Early stages of fishes in the Western North Atlantic Ocean*. NAFO. From Moser, Charter. 1996a | fishbase: Noche_u1.jpg by Dolgov A. |
| *Albula vulpes* | 17.24,  9.07 | fishbase: Alvule_10.jpg by Sazima I. | MAB I: Goode GB, and a Staff of Associates. 1884 |
| *Eurypharynx pelecanoides* | 3.27,  30.67 | Miller MJ. 2004 An Introduction to Leptocephali Biology and Identification. Ocean Research Institute, University of Tokyo | NMNZ P. 38952 by McPhee R. |
| *Anguilla japonica* | 16.06,  22.30 | Miller MJ. 2009 Ecology of Anguilliform leptocephali: remarkable transparent fish larvae of the ocean surface layer. Aqua-*BioSci. Monogr*. 2(4),1-94 | fishbase: Anjap_u0.jpg Shao KT. |
| *Anguilla rostrata* | 8.62,  13.68 | Fahay MP. 2007 *Early stages of fishes in the Western North Atlantic Ocean*. redrawn from Vladykov. 1955 | [http://txstate.fishesoftexas.org/anguilla%20rostrata.htm: photo by Thomas](http://txstate.fishesoftexas.org/anguilla%20rostrata.htm:%20photo%20by%20Thomas) C. |
| *Gymnothorax funebris* | 9.14,  12.97 | MAB II: Eldred B. 1970 | fishbase: Gyfun_u0.gif Ref No 9358 |
| *Conger oceanicus* | 13.03,  19.73 | Bell GW, Witting, DA, and Able, KW. 2003 Aspects of metamorphosis and habitat use in the Conger Eel, Conger oceanicus. *Copeia*. 3, 544-552 | MAB VII: Bigelow HB, Schroeder WC. 1953 |
| *Hiodon alosoides* | 15.91,  3.84 | Battle HI, Sprules WM. 1960 A description of the semi-buoyant eggs and early stages of the goldeye, hiodon-alosoides (Rafinesque)  *J Fish Board Can*. 17(2), 245-266. | Stewart K, Watkinson D. 2004 *Freshwater fishes of Manitoba*. Univ. of Manitoba Press |
| *Hiodon tergisus* | 15.40,  3.71 | Snyder DE, Douglas SC. 1987 Description and identification of mooneye, Hiodon tergisus, protolarvae, *T Am Fish Soc*. 107(4), 590-594 | Stewart K, Watkinson D. 2004 *Freshwater fishes of Manitoba*. Univ. of Manitoba Press |
| *Osteoglossum bicirrhosum* | 12.08,  5.18 | Yanwirsal H. 2013 Reproductive styles of Osteoglossomorpha with emphasis on *Notopterus notopterus* and *Osteoglossum bicirrhosum* (Doctoral dissertation, Humboldt-Universität zu Berlin, Landwirtschaftlich-Gärtnerische Fakultät) | fishbase: Osbic_u0.jpg by IBAMA |
| *Petrocephalus soudanensis* | 12.71,  3.45 | Kirschbaum F, Schugardt C. 2002 Reproductive strategies and developmental aspects in mormyrid and gymnotiform fishes. *J Physiol*. 96, 557-566 | Moritz T, Engelmann J, Linsenmair KE, von der Emde G. 2009 The electric organ discharges of the *Petrocephalus*species (Teleostei: Mormyridae) of the upper volta system. *J Fish Biol*. 74, 54-76 |
| *Notopterus notopterus* | 10.36,  3.86 | Termvidchakorn A, Hortle KG. 2013 A guide to larvae and juveniles of some common fish species from the Mekong River Basin. MRC Technical Paper No. 38. Mekong River Commission, Phnom Penh. 234pp. | fishbase: Notopterus_notopterus.jpg by Mahalder B |
| *Chitala ornata* | 8.30,  4.14 | Termvidchakorn A, Hortle KG. 2013 A guide to larvae and juveniles of some common fish species from the Mekong River Basin. MRC Technical Paper No. 38. Mekong River Commission, Phnom Penh. 234pp. | fishbase: Chorn_u4.jpg by Baird IG. |
| *Dorosoma petenense* | 22.84,  4.18 | MAB I: Taber CA. 1961 | <http://txstate.fishesoftexas.org/dorosoma%20petenense.htm> |
| *Anchoa hepsetus* | 37.03,  5.69 | MAB I: Hildebrand SF. 1963b | fishbase: Anhep_u0.jpg by Flescher D. |
| *Gonorynchus greyi* | 17.29,  13.25 | Leis JM, Trnski T, Bruce B. 1989 *The larvae of Indo-Pacific shorefishes*. Honolulu: University of Hawaii Press. | fishbase: Gogre_u9.jpg by Randall JE. |
| *Chanos chanos* | 18.82,  6.93 | Bagarinao TU. 1991 Biology of milkfish (Chanos chanos Forsskal). Iloilo, Philippines: Aquaculture Department, Southeast Asian Fisheries Development Center | fishbase: Chcha_u2.jpg by Randall JE |
| *Carassius auratus* | 16.62,  3.93 | Battle HI. 1940 The embryology and larval development of the goldfish (*Carassius Auratus L*.) from Lake Erie. *Ohio J Sci*. 40(2), 82-93 | fishbase: Caaurau1.jpg by Winter TJ |
| *Cyprinus carpio* | 11.75,  4.15 | Cole NJ, Hall TE, Martin CI, Chapman MA, Kobiyama A, Nihei Y, Watabe S, and Johnston IA. 2004 Temperature and the expression of myogenic regulatory factors (MRFs) and myosin heavy chain isoforms during embryogenesis in the common carp Cyprinus carpio L. *JEB*. 207, 4239-4248 | fishbase: Cyprinus.jpg by Kibria M, Asma GSM, Arafeen S. |
| *Danio rerio* | 12.96,  6.30 | Kimmel CB, Ballard WW, Kimmel SR, Ullmann B, and Schilling TF. 1995 Stages of Embryonic development of the zebrafish. *Dev Dynam*. 203, 253-310 | photo by Hilary Katz |
| *Catostomus commersonii* | 17.55,  6.79 | Fishbase: Cacom_I0.gif by Faber DJ | http://gallery.nanfa.org/v/members/Uland/Family+Catostomidae/Catostomus/Catostomus+commersoni++White+Sucker+.jpg.html?g2_imageViewsIndex=1 |
| *Erimyzon oblongus* | 12.47,  7.02 | Fuiman LA. 1979 Descriptions and Comparisons of Catostomid Fish Larvae: Northern Atlantic Drainage Species, *T Am Fish Soc*. 108(6), 560-603, | MAB I: Trautman MB. 1957 |
| *Eigenmannia lineata* | 10.29,  8.10 | Kirschbaum F, Schugardt C. 2002 Reproductive strategies and developmental aspects in mormyrid and gymnotiform fishes. *J Physiol - Paris*. 96, 557-566 | Kirschbaum F, Schugardt C. 2002 Reproductive strategies and developmental aspects in mormyrid and gymnotiform fishes. *J Physiol - Paris*. 96:557-566 |
| *Apteronotus leptorhynchus* | 12.47,  6.51 | Kirschbaum F, Schugardt C. 2002 Reproductive strategies and developmental aspects in mormyrid and gymnotiform fishes. *J Physiol - Paris*. 96, 557-566 | Kirschbaum F, Schugardt C. 2002 Reproductive strategies and developmental aspects in mormyrid and gymnotiform fishes. *J Physiol - Paris*. 96, 557-566 |
| *Hypophthalmus edentatus* | 12.66,  4.72 | Nakata K, Baumgartner G, Latini JD. 1998 Morphological description of larvae of the mapara *Hypophthalmus edentatus* (spix) (Osteichthyes, Hypophthalmidae) in the Itaipu reservoir (Parana river, Brazil) | fishbase: H_edentatus.jpg by Ferreira ES. |
| *Ictalurus punctatus* | 10.00,  5.73 | MAB I: Greeley JR, Bishop SC. 1932 | MAB I: Trautman MB. 1957 |
| *Arius felis* | 8.22,  8.07 | MAB I: Mansueti AJ, Hardy JD. 1967 | fishbase: Arfel_u0.jpg by Flescher D. |
| *Pygocentrus nattereri* | 12.43,  2.14 | Bender A, Moritz T. 2013 Developmental residue and developmental novelty - different modes of adipose-fin formation during ontogeny. *Zoosyst. Evol.* 89(2), 209-214 | fishbase: Pynat_u9.jpg by Timm CD. |
| *Salminus brasiliensis* | 16.29,  4.83 | Santos JE, Godinho HP. 2002 Ontogenic events and swimming behavior of larvae of the characid fish Salminus brasiliensis (Cuvier) (Characiformes, Characidae) under laboratory conditions. *Revta. bras. Zool*. 19(1), 163-171 | fishbase: Sabra_u6.jpg by Sverlij S. |
| *Salmo salar* | 12.31,  7.99 | Gorodilov YN. 1996 Description of the early ontogeny of the Atlantic salmon, *Salmo salar*, with a novel system of interval (state) identification, *Env. Biol. Fish.* 47:109-127 | fishbase: Sasal_u5.jpg by McDowall RM. |
| *Esox americanus* | 16.15,  6.65 | Leslie JK, Gorrie JF, 1985 Distinguishing features for separating protolarvae of three species of esocids. *Can. Tech. Rep. Fish. Aquat. Sci.* 1359:82 p. | MAB I: Mansueti AJ and Hardy JD. 1967 |
| *Argyropelecus hemigymnus* | 17.72,  5.29 | Richards WJ. (Ed.). 2004 Early stages of Atlantic fishes: an identification guide for the western central north Atlantic, Two Volume Set. CRC Press. | fishbase: Arhem_u6.jpg by Fischer LG. |
| *Cyclothone braueri* | 15.16,  11.43 | Fahay MP. 2007 *Early stages of fishes in the Western North Atlantic Ocean*. NAFO. From Sanzo 1931a | MCZbase: The database of zoological collections: Cat Num: 143600 |
| *Osmerus mordax* | 25.56,  9.26 | Cooper JE. 1978 Identification of eggs, larvae, and juveniles of the rainbow smelt, *Osmerus mordax*, with comparisons to larval alewife, *Alosa pseudoharengus*, and gizzard shad, *Dorosoma cepedianum*. *T Am Fish Soc*, 107(1), 56-62 | Osmor_u2.jpg by Lyons J. |
| *Microstoma microstoma* | 23.47,  13.16 | Fahay MP. 2007 *Early stages of fishes in the Western North Atlantic Ocean*. NAFO. From Olivar and Fortuno 1991, redrawn from Sanzo, 1931a | fishbase: mimic_u0.gif Ref. No. 3978 |
| *Ateleopus japonicus* | 28.67,  11.07 | Amaoka K. 2003 *Preliminary Guide to the Identification of the Early Life History Stages of Ateleopodid Fishes of the Western Central North Atlantic*. US Department of Commerce, NOAA, NMFS, Southeast Fisheries Science Center. | fishbase: Atjap_u3.jpg by Ho HC. |
| *Arctozenus risso* | 17.26,  15.48 | Richards WJ. (Ed.) 2004 Early stages of Atlantic fishes: an identification guide for the western central north Atlantic, Two Volume Set. CRC Press. From Ege 1930 | fishbase: Arris_u0.jpg by Dolgov. |
| *Synodus foetens* | 14.53,  11.40 | Richards WJ. (Ed.) 2004 Early stages of Atlantic fishes: an identification guide for the western central north Atlantic, Two Volume Set. CRC Press. From Able and Fahay 1998 | fishbase: syfoe_u0.jpg by Flescher D. |
| *Electrona risso* | 13.98,  3.84 | Richards, W. J. (Ed.). 2004 Early stages of Atlantic fishes: an identification guide for the western central north Atlantic, Two Volume Set. CRC Press. | fishbase: Elris_u1.jpg by Costa F. |
| *Neoscopelus macrolepidotus* | 7.26,  5.84 | Richards, W. J. (Ed.). 2004 Early stages of Atlantic fishes: an identification guide for the western central north Atlantic, Two Volume Set. CRC Press. From Okiyama 1988c | fishbase: Nemac_u1.jpg by JAMARC |
| *Polymixia lowei* | 2.66,  3.90 | Fahay MP. 2007 *Early stages of fishes in the Western North Atlantic Ocean*. NAFO. From Bond PJ (Lyczkowski-Shultz 2006) | fishbase: Polow_u0.jpg by Flescher D. |
| *Aphredoderus sayanus* | 10.54,  4.54 | Minton AL, Osteen DV, Snyder DE. 1985 Description of larval pirate perch, *Aphredoderus sayanus* (Gilliams), from the Savannah River. Can. Tech. Rep. Fish. Aquat. Sci. 1359:82 p. | fishbase: Apsay_f0.jpg by Burkhead N and Jenkins R |
| *Zeus faber* | 8.26,  1.94 | fishbase: Zefab_I1.gif Ref. No. 63 | fishbase: Zefab_f0.jpg by Cambraia Duarte PMN |
| *Zenopsis conchifera* | 9.08,  2.27 | Fahay MP. 2007 *Early stages of fishes in the Western North Atlantic Ocean*. NAFO. From Weiss et al. 1987 | fishbase: Zecon_f0.jpg by Cambraia Duarte PMN. |
| *Merluccius australis* | 13.28,  9.16 | Bustos CA, Landaeta MF. 2005 Development of eggs and early larvae of the Southern hake, *Merluccius australis* reared under laboratory conditions. *Gayana*. *69*, 402-408 | fishbase: Meaus_u0.jpg by SeaFIC. |
| *Merluccius bilinearis* | 15.60,  5.81 | MAB II: Kuntz A, Radcliffe L. 1918 | fishbase: Mebil_u0.jpg by Flescher D. |
| *Urophycis chuss* | 10.39,  6.25 | fishbase: from Scotton, L.N., R.E. Smith, N.S. Smith, K.S. Price and D.P. de Sylva, 1973. Pictorial guide to fish larvae of Delaware Bay: with information and bibliographies useful for the study of fish larvae. Delaware Bay Report Series. Vol. 7. College of Marine Studies, University of Delaware. 205 p. | MAB II: Goode GB. 1884 |
| *Lota lota* | 12.20,  7.91 | Palińska-Żarska K, Żarski D, Krejszeff S, Nowosad J, Biłas M, Trejchel K, Kucharczyk D. 2014 Dynamics of yolk sac and oil droplet utilization and behavioural aspects of swim bladder inflation in burbot, *Lota lota* L., larvae during the first days of life, under laboratory conditions. *Aquacul Int*. *22*(1), 13-27 | fishbase: Lota_ua.jpg by Artaev O. |
| *Brosme brosme* | 8.34,  4.74 | fishbase: Brbro_I1.gif by Faber DJ. | fishbase: Brbro_u0.jpg by Flescher D. |
| *Pollachius virens* | 14.91,  6.05 | Scotton LN, Smith RE, Smith NS, Price KS, de Sylva DP. 1973 Pictorial guide to fish larvae of Delaware Bay. Univ. Delaware, Del. Bay Rept. Ser, 7, 205: Bigelow and Schroeder, 1953 | Povir_u0.jpg by Flescher D. |
| *Gadus morhua* | 15.65,  5.20 | Norman T Nicoll/ Natural Visions, image reference: NN_86_01_15 | fishbase: Gamor_ub.jgp by Nilsson K. |
| *Lophotus lacepede* | 11.40,  20.65 | Fahay MP. 2007 *Early stages of fishes in the Western North Atlantic Ocean*. NAFO. From Sanzo 1940b | Lolac_u0.jpg by Ragonese S. |
| *Lampris guttatus* | 6.81,  1.70 | Ahlstrom EH. 1984 Ontogeny and systematics of fishes: Based on an International Symposium Dedicated to the Memory of Elbert Halvor Ahlstrom. American Society of Ichthyologists and Herpetologists. MCZ 58990 | fishbase: 6760_full_107cm.jpg by Raulsinho R. |
| *Beryx splendens* | 15.54,  3.40 | Fahay MP. 2007 *Early stages of fishes in the Western North Atlantic Ocean*. NAFO. From Munday, 1990 | fishbase: Bespl_f0.jpg by Duarte C, P.M.N. |
| *Anoplogaster cornuta* | 2.74,  3.74 | Fahay MP. 2007 *Early stages of fishes in the Western North Atlantic Ocean*. NAFO. From Richards et al., 2003 | fishbase: Ancor_u1.jpg by Diaz B. |
| *Barbourisia rufa* | 17.64,  4.54 | Paxton JR, Johnson GD, Trnski T. 2001 Larvae and juveniles of the deepsea "whalefishes" Barbourisia and Rondeletia (Stephanoberyciformes: Barbourisiidae, Rondeletiidae), with comments on family relationships. *Records-Australian Museum*, 53(3), 407-426 | fishbase: Baruf_u0.jpg by JAMARC |
| *Rondeletia loricata* | 4.89,  3.46 | Paxton JR, Johnson GD, Trnski T. 2001 Larvae and juveniles of the deepsea" whalefishes" Barbourisia and Rondeletia (Stephanoberyciformes: Barbourisiidae, Rondeletiidae), with comments on family relationships. *Records-Australian Museum*, 53(3), 407-426 | fishbase: Rolor_u0.jpg by Garazo Fabregat A. |
| *Poromitra megalops* | 5.47,  5.32 | Fahay, M. P. (2007). *Early stages of fishes in the Western North Atlantic Ocean*. NAFO. From Sandknop and Watson, 1996a | fishbase: Pomeg_u0.jpg by Garazo Fabregat A./ Roman Marcote E. |
| *Otophidium omostigma* | 13.55,  5.70 | Richards WJ. (Ed.) 2004 Early stages of Atlantic fishes: an identification guide for the western central north Atlantic, Two Volume Set. CRC Press. From Jordan & Gilbert 1882 | fishbase: Otomo_u0.jpg by JAMARC |
| *Ophidion marginatum* | 14.80,  8.19 | MAB V: Scotton LN, etal. 1973 | fishbase: Opmar_u0.jpg by Flescher D. |
| *Porichthys notatus* | 7.00,  5.05 | Richards WJ. (Ed.) 2004 Early stages of Atlantic fishes: an identification guide for the western central north Atlantic, Two Volume Set. CRC Press. From Watson 1996f | fishbase: Ponot_u0.gif, Ref. No. 12204 |
| *Opsanus tau* | 10.07,  5.24 | MAB VI: Dovel W. 1960 | MAB VI: Bigelow HB, Schroeder WC. 1953 |
| *Macroramphosus scolopax* | 7.71,  2.89 | Kuranaga I, Sasaki K. 2000 Larval development in a snipefish (*Macroramphosus scoplopax*) from Japan with notes on eastern Pacific and Mediterranean Macroramphosus larvae (*Gasterosteiformes, Macroramphosidae*). *Ichthyol Res*. 47(1), 101-106 | [http://australianmuseum.net.au/image/A-Common-Bellowsfish-trawled-off-Norah-Head/ by Ken Graham](http://australianmuseum.net.au/image/A-Common-Bellowsfish-trawled-off-Norah-Head/%20by%20Ken%20Graham) |
| *Fistularia petimba* | 28.47,  37.39 | Barros FBAGD, Castro MSD, Bonecker ACT. 2007 Description and distribution of the larvae of two species of Fistulariidae (Teleostei, Syngnathiformes) in the southeastern Brazil. *Biota Neotropica*, 7(1) | fishbase: Fipet_u2.jpg by Randall JE. |
| *Paralichthys dentatus* | 13.25,  3.21 | Martinez GM, Bolker JA. 2003 Embryonic and larval staging of summer flounder (*Paralichthys dentatus*). *J Morphol*. 255(2), 162-176 | fishbase: Paden_u0.jpg by Flescher D. |
| *Syacium papillosum* | 8.52,  2.88 | MAB VI: Futch CR, Hoff FH Jr., 1971 | fishbase: Sypap_u2.jpg by NOAA/NMFS/Mississippi Laboratory |
| *Synbranchus lampreia* | 29.63,  24.23 | Favorito SE, Zanata AM, Assumpção MI. 2005 A new Synbranchus (Teleostei: Synbranchiformes: Synbranchidae) from ilha de Marajó, Pará, Brazil, with notes on its reproductive biology and larval development. *Neotrop Ichthyol*. 3(3), 319-328 | Favorito SE, Zanata AM, Assumpção MI. 2005 A new Synbranchus (Teleostei: Synbranchiformes: Synbranchidae) from ilha de Marajó, Pará, Brazil, with notes on its reproductive biology and larval development. *Neotrop Ichthyol*. *3*(3), 319-328 |
| *Gobiesox strumosus* | 11.62,  6.86 | Fahay MP. 2007 *Early stages of fishes in the Western North Atlantic Ocean*. NAFO. From Runyan, 1961 | fishbase: Gostr_u0.gif Ref. No. 9358 |
| *Cololabis saira* | 15.38,  9.33 | fishbase: Cosai_I0.gif, Ref. No. 265 | fishbase: CosaiU0.jpg by Miyahara H. |
| *Hemiramphus brasiliensis* | 15.53,  10.95 | Hardy JD, Johnson RK. 1974 Descriptions of halfbeak larvae and juveniles from Chesapeake Bay (Pisces: Hemiramphidae). *Chesapeake Science*. *15*(4), 241-246. | MAB II: Jordan DS, Evermann BW. 1896-1900 |
| *Cheilopogon cyanopterus* | 6.73,  6.85 | Fahay MP. 2007 *Early stages of fishes in the Western North Atlantic Ocean*. NAFO. From T.N. Steyker (Kovalevskaya, 1977) | fishbase: Chcya_u3.jpg by Hermosa GV Jr. |
| *Tylosurus acus melanotus* | 16.43,  11.47 | Fahay MP. 2007 *Early stages of fishes in the Western North Atlantic Ocean*. NAFO. From Mito, 1958 (redrawn) | fishbase: Tyacu_u0.jpg by Shao KT. |
| *Ablennes hians* | 20.63,  11.93 | Fahay MP. 2007 *Early stages of fishes in the Western North Atlantic Ocean*. NAFO. From Chen 1988 | MAB II: Bigelow HB, Schroeder WC. 1953 |
| *Fundulus diaphanus* | 9.56,  5.84 | Jones GG, Tabery MA, Turnpike G. 1980 Larval development of the banded killifish (*Fundulus diaphanus*) with notes on the distribution in the Hudson River estuary. In *Proceedings of the Fourth Annual Larval Fish Conference, February 27-28, 1980, Oxford, Mississippi* (p. 25). Fish and Wildlife Service, US Department of the Interior. | fishbase: Fudia_f0.jpg by Burkhead N, Jenkins R, courtesy of VDGIF |
| *Lucania parva* | 10.39,  4.28 | Crawford SS, Balon EK. 1994 Alternative life histories of the genus Lucania: 1. Early ontogeny of *L. parva*, the rainwater killifish. *Environ Biol Fishes*. 40(4), 349-389 | MAB II: Bean TH. 1888 |
| *Atherina presbyter* | 11.50,  6.00 | Bamber RN, Henderson PA, Turnpenny AWH. 1985 The early life history of the sand smelt (*Atherina presbyter*). *J Mar Biol Assoc UK,* 65(03), 697-706. | fishbase: MNHN 2004-1491 |
| *Membras martinica* | 18.65,  7.29 | MAB VI: Wang JCS. 1974 | fishbase: Memar_u1.jpg by Thomas C. |
| *Dactylopterus volitans* | 9.41,  8.15 | Fahay MP. 2007 *Early stages of fishes in the Western North Atlantic Ocean*. NAFO. From Padoa 1956c | fishbase: Davol_u3.jpg by Cambraia Duarte, PMN. |
| *Hemitripterus americanus* | 12.23,  5.57 | Fahay MP. 2007 *Early stages of fishes in the Western North Atlantic Ocean*. NAFO. From Fuiman 1976 | MAB V: Goode GB. 1884 |
| *Eurypegasus draconis* | 13.37,  9.72 | Herold D, Clark E. 1993 Monogamy, spawning and skin-shedding of the sea moth, *Eurypegasus draconis* (Pisces: Pegasidae). *Environ Biol Fishes*. 37(3), 219-236 | fishbase: Eudra_u7 by Randall JE. |
| *Gasterosteus aculeatus* | 12.67,  6.79 | fishbase: Gaacu_I0.jpg by Pinder AC. | fishbase: Gaacu_u7.jpg by Miyahara H. |
| *Sphoeroides maculatus* | 7.95,  6.91 | MAB VI: Welsh WW, Brender CM Jr. 1922 | fishbase: Spmac_u0.jpg by Flescher D. |
| *Balistes capriscus* | 5.91,  2.03 | Matsuura Y, Katsuragawa M. 1981 Larvae and juveniles of grey triggerfish, *Balistes capriscus*, from Southern Brazil. *Jap J Ichthyol*. 28(3), 267-275 | fishbase: Bacar_u1.jpg by Flescher D. |
| *Chaenophryne longiceps* | 3.49,  2.88 | Fahay MP. 2007 *Early stages of fishes in the Western North Atlantic Ocean*. NAFO. From Bertelsen, 1951 | Australian Museum CSIRO H6022-01 |
| *Antennarius pauciradiatus* | 4.19,  3.52 | Baldwin CC. 2013 The phylogenetic significance of colour patterns in marine teleost larvae. *Zool J of Linnean Soc*. 168(3), 496-563 | Williams JT, Carpenter KE, Van Tassell JL, Hoetjes P, Toller W, Etnoyer P, Smith M. 2010 Biodiversity assessment of the fishes of Saba Bank atoll, Netherlands Antilles. *PloS one*. 5(5), e10676 |
| *Histrio histrio* | 12.67,  3.15 | MAB VI: Fujita S, Uchida K. 1959 | fishbase: Hihis_u5.jpg by Randall JE. |
| *Trichiurus lepturus* | 13.08,  13.59 | fishbase: Trlep_I3.gif Ref No. 44342 | fishbase: Trlep_u1.jpg by Flescher D. |
| *Lepidopus caudatus* | 15.98,  14.38 | fishbase: Lecau_I0.gif, Ref. No. 29072 | fishbase: Lecau_f0.jpg by Cambraia Duarte, PMN. |
| *Lepidocybium flavobrunneum* | 4.02,  4.96 | Nishikawa Y. 1982 Early development of the fishes of the family Gempylidae I. Larvae and juveniles of escolar, *Lepidocybium flavobrunneum* (Smith). *Bull Far Seas Fish Res Lab*. 19, 1-19. | fishbase: Lefla_u1.jpg by Camraia Duarte, PMN. |
| *Tetragonurus atlanticus* | 16.64,  7.42 | MAB VI: Ahlstrom EH, Butler JL, and Sunida BY. 1976 | fishbase: Teatl_u0.gif, Ref. No. 4415 |
| *Peprilus paru* | 12.92,  1.70 | MAB VI: Pearson JC. 1941 | fishbase: Pepar_u1.jpg by Ramjohn DD. |
| *Scomber scombrus* | 14.36,  6.29 | fishbase: Scsco_I0.jpg by Mendiola D. | fishbase: NRM 49614b from Swedish Museum of Natural History |
| *Euthynnus affinis* | 5.32,  4.95 | National Bioresource Development Board, Dept. of Biotechnology, Government of India, New Delhi. Reproduced from Gorbunova, 1974 | fishbase: Euaff_u1.jpg by Randall JE. |
| *Lepomis cyanellus* | 10.24,  2.77 | Taubert BD. 1977 Early morphological development of the green sunfish, *Lepomis cyanellus*, and its separation from other larval Lepomis species. *T Am Fish Soc*. 106(5), 445-448 | fishbase: Lecya_m6.jpg by Burkhead N, Jenkins R, courtesy of VDGIF |
| *Epinephelus niveatus* | 11.92,  3.49 | Powell AB, Tucker JW. 1992 Egg and larval development of laboratory-reared Nassau grouper, *Epinephelus striatus* (Pisces, Serranidae). *Bull Mar Sci*. 50(1), 171-185 | fishbase: Epniv_u0.jpg by Flescher D. |
| *Sphyraena borealis* | 11.65,  8.34 | MAB VI: Houde ED. 1972 | fishbase: Spbor_u0.jpg by Flescher D. |
| *Sphyraena tome* | 14.85,  7.40 | Matsuura Y, Suzuki K. 1997 Larval development of two species of barracuda, *Sphyraena guachancho* and *S. tome* (Teleostei: Sphyraenidae), from southeastern Brazil. *Ichthyol Res*. 44(4), 369-378 | fishbase: sphyraenatome.jpg by Vaske T Jr. |
| *Mugil curema* | 9.18,  5.37 | MAB VI: Anderson WW. 1957 | fishbase: Mucur_u1.jpg by CENAIM |
| *Perca flavescens* | 10.57,  4.81 | Mansueti AJ. 1964 Early development of the yellow perch, *Perca flavescens*. *Chesapeake Science*. 5(1-2), 46-66 | fishbase: Pefla_m0.jpg by Burkhead N, Jenkins R, courtesy of VDGIF |
| *Lobotes surinamensis* | 7.79,  2.74 | Ditty JG, Shaw RF. 1994 *Lobotes surinamensis* (Pisces: Lobotidae), and their spatial and temporal distribution in the northern Gulf of Mexico. *Fish Bull*. 92(1), 33-45 | fishbase: Losur_u0.jpg by Jimenez PP. |
| *Pomacanthus rhomboides* | 11.98,  1.79 | Baldwin CC. 2013 The phylogenetic significance of colour patterns in marine teleost larvae. *Zool J Linnean Soc*. 168(3), 496-563 | fishbase: Porho_u0.jpg by Randall JE. |
| *Ocyurus chrysurus* | 7.39,  3.27 | Riley CM, Holt GJ, Arnold CR. 1995 Growth and morphology of larval and juvenile captive bred yellowtail snapper, *Ocyurus chrysurus*. *Fish Bull*. *93*, 179-185 | fishbase: Occur_u8.jpg by Macieira RM. |
| *Lutjanus griseus* | 13.36,  3.38 | Richards WJ, Saksena VP. 1980 Description of larvae and early juveniles of laboratory-reared gray snapper, *Lutjanus griseus* (Linnaeus)(Pisces, Lutjanaidae). *Bull Mar Sci*. *30*(2), 515-521 | fishbase: Lugri_u0.jpg by Flescher D. |
