# Supplementary material for: A Large-Scale Pattern of Ontogenetic Shape Change in Ray-Finned Fishes
Source: PLoS One. 2016 Mar 4;11(3):e0150841. doi: 10.1371/journal.pone.0150841 (PMC4778928; doi:10.1371/journal.pone.0150841)
Supplement: S2 Table — These values were not included in the statistical analyses since we were not able to find a second source for every study. We chose to include these sources for the benefit of individuals looking to find multiple online sources for larval and adult stages. (DOCX) [file pone.0150841.s002.docx]

**S2 Table: Secondary values and sources**. These values were not included in the statistical analyses since we were not able to find a second source for every study. We chose to include these sources for the benefit of individuals looking to find multiple online sources for larval and adult stages.

| *Species* | larval  adult elongation | larval source | adult source |
| --- | --- | --- | --- |
| *Acipenser oxyrhyncus* | 15.00,  x | MAB I: Ryder JA. 1890 | x |
| *Lepisosteus osseus* | x,  13.82 | x | Official gallery of NANFA: Longnose gar 1500 |
| *Amia calva* | x,  5.93 | x | <http://txstate.fishesoftexas.org/amia%20calva.htm> |
| *Elops saurus* | x,  9.23 | x | NOAA photo library: fish4201 by Noble B |
| *Megalops atlanticus* | x,  x | x | x |
| *Notacanthus chemnitzii* | x,  x | x | x |
| *Albula vulpes* | 35.95,  7.91 | MAB I: Alexander EC. 1961 | Marinebio.org species database |
| *Eurypharynx pelecanoides* | x,  x | x | x |
| *Anguilla japonica* | 14.81,  x | Miller MJ. 2004 An Introduction to Leptocephali Biology and Identification. Ocean Research Institute, University of Tokyo | x |
| *Anguilla rostrata* | x,  x | x | x |
| *Gymnothorax funebris* | x,  17.26 | x | fishbase: Gyfun_u4.jpg by Hofinger E. |
| *Conger oceanicus* | 17.92,  x | MAB VII: Bigelow HB, Schroeder WC. 1953 | x |
| *Hiodon alosoides* | x,  x | x | x |
| *Hiodon tergisus* | x,  4.05 | x | ODNR Division of Wildlife by Zimmerman B |
| *Osteoglossum bicirrhosum* | x,  x | x | x |
| *Petrocephalus soudanensis* | x,  2.84 | x | Moritz T, Engelmann J, Linsenmair KE, von der Emde G. 2009 The electric organ discharges of the *Petrocephalus* species (Teleostei: Mormyridae) of the upper volta system. *J Fish Biol*. 74, 54-76 |
| *Notopterus notopterus* | x,  x | x | x |
| *Chitala ornata* | x,  x | x | x |
| *Dorosoma petenense* | x,  4.78 | x | MAB I: Miller RR. 1963 |
| *Anchoa hepsetus* | x,  7.05 | x | MAB I: Hildebrand SF, Cable LE. 1930 |
| *Gonorynchus greyi* | x,  x | x | x |
| *Chanos chanos* | 19.00,  7.41 | fishbase: Chcha_I0.gif, Ref No 41564 | fishbase: Chcha_u0.jpg by Shao KT. |
| *Carassius auratus* | x  x | x | x |
| *Cyprinus carpio* | 17.67,  5.60 | MAB I: Taber CA. 1969 | MAB I: Smith HM. 1896 |
| *Danio rerio* | x,  x | x | x |
| *Catostomus commersonii* | x,  x | x | x |
| *Erimyzon oblongus* | 19.47,  6.20 | MAB I: Carnes WC Jr. 1958 | fishbase: Erbol_u1.jpg by Lyons J. |
| *Eigenmannia lineata* | x,  8.20 | x | fishbase: Eivir_u4.jpg by Holm E. |
| *Apteronotus leptorhynchus* | x  x | x | x |
| *Hypophthalmus edentatus* | x  4.72 | x | fishbase: MNHN A-896A |
| *Ictalurus punctatus* | x,  6.57 | x | mid-atlantic stocking: http://www.midatlanticstocking.com/images/Channel%20Catfish.gif |
| *Arius felis* | x,  8.52 | x | MAB I: Goode GB, etal. 1884 |
| *Pygocentrus nattereri* | x,  2.44 | x | fishbates: Pynat_u3.jpg by Ostergaard T |
| *Salminus brasiliensis* | x,  4.82 | x | fishbase: Sabra_ub.jpg by Timm CD. |
| *Salmo salar* | 10.61,  x | von Schalburg KR, Yasuike M, Yazawa R, De Boer JG, Reid L, So S, Robb A, Rondeau EB, Phillips RB, Davidson WS, Koop BF. 2011. Regulation and expression of sexual differentiation factors in embryonic and extragonadal tissues of Atlantic salmon. *BMC genomics*. 12(1), 31 | x |
| *Esox americanus* | 16.00,  x | MAB I: Mansueti AJ, Hardy JD Jr, 1967 | x |
| *Argyropelecus hemigymnus* | x,  4.94 | x | fishbase: Arhem_u1.jpg by Costa F. |
| *Cyclothone braueri* | x,  11.87 | x | fishbase: Cybra_u0.jpg by Costa F. |
| *Osmerus mordax* | x,  x | x | x |
| *Microstoma microstoma* | x,  12.08 | x | fishbase: Mimic_u1.jpg by Costa F. |
| *Ateleopus japonicus* | x,  19.06 | x | Amaoka K. 2003 *Preliminary Guide to the Identification of the Early Life History Stages of Ateleopodid Fishes of the Western Central North Atlantic*. US Department of Commerce, NOAA, NMFS, SFSC. |
| *Arctozenus risso* | x,  x | x | x |
| *Synodus foetens* | 16.74,  13.55 | MAB I: Mansueti AJ, Hardy JD Jr. 1967 | MAB I: Anderson WW, Gehringer JW, Berry FH. 1966 |
| *Electrona risso* | x,  3.35 | x | fishbase: Elris_u0.jpg by JAMARC |
| *Neoscopelus macrolepidotus* | x,  6.33 | x | World Register of Marine Species: Noères, Claude |
| *Polymixia lowei* | x,  4.02 | x | fishbase: Polow_u1.gif Ref. No. 9358 |
| *Aphredoderus sayanus* | 15.26,  5.17 | MAB III: Drawn from photographs, Peters ER | MAB III: Trautman MB. 1957 |
| *Zeus faber* | x,  2.15 | x | fishbase: Zefab_uc.jpg by Dijkstra K. |
| *Zenopsis conchifera* | x,  1.87 | x | fishbase: PA034125.jpg by Winkler M. |
| *Merluccius australis* | 15.30,  x | fishbase: Meaus_I1.jpg by Chile F. | x |
| *Merluccius bilinearis* | 9.32,  6.20 | fishbase: Mebil_I0.gif by Faber DJ | MAB II: Goode GB. 1884 |
| *Urophycis chuss* | x,  x | x | x |
| *Lota lota* | x,  x | x | x |
| *Brosme brosme* | x,  x | x | x |
| *Pollachius virens* | x,  x | x | x |
| *Gadus morhua* | 13.34,  4.39 | MAB II: M'Intosh WC, Masterman AT. 1897 | MAB II: Goode GB. 1884 |
| *Lophotus lacepede* | x,  20.65 | x | Dulčić, J, Ahnelt H. 2007 How many specimens of the crested oarfish, *Lophotus lacepede* *Giorna*, 1809 (Pisces: Lophotidae), were caught in the Adriatic Sea? *Acta adriatica*. 48(1), 39-43. |
| *Lampris guttatus* | x,  1.86 | x | fishbase: Lagut_ua.jpg by Mincarone MM |
| *Beryx splendens* | x,  4.84 | x | fishbase: Bespl_ud.jpg by Labbe J, Ahumada M. |
| *Anoplogaster cornuta* | x,  3.98 | x | fishbase: Ancor_u0.jpg by JAMARC |
| *Barbourisia rufa* | x,  5.44 | x | fishbase: Baruf_u0.gif, Ref. No. 4249 |
| *Rondeletia loricata* | x,  2.74 | x | fishbase: Rolor_u0.gif, Ref. No. 4246 |
| *Poromitra megalops* | x,  4.47 | x | fishbase: Pomeg_u0.gif, Ref. No. 4241 |
| *Otophidium omostigma* | x,  5.28 | x | eol.org: Cat num 167777 from Museum of Comparative Zoology, Harvard |
| *Ophidion marginatum* | x,  8.21 | x | MAB V: Hildebrand SF, Schroeder WC. 1928 |
| *Porichthys notatus* | x,  x | x | x |
| *Opsanus tau* | x,  x | x | x |
| *Macroramphosus scolopax* | 14.03,  3.25 | MAB II: Sparta A. 1936 | MAB II: Kamohara T. 1967 |
| *Fistularia petimba* | 28.08,  30.21 | MAB II: Mito S. 1961 | fishbase: Fipet_u3.jpg by Cambraia Duarte, PMN |
| *Paralichthys dentatus* | 12.38,  3.41 | MAB VI: Smith WG, Fahay MP. 1970 | MAB VI: Norman JR. 1934 |
| *Syacium papillosum* | x,  2.99 | x | MAB VI: Futch CR, Hoff FH Jr. 1971 |
| *Synbranchus lampreia* | x,  x | x | x |
| *Gobiesox strumosus* | x,  x | x | x |
| *Cololabis saira* | x,  x | x | x |
| *Hemiramphus brasiliensis* | 14.73,  x | MAB II: Hardy JD, Johnson RK. 1974 | x |
| *Cheilopogon cyanopterus* | x,  x | x | x |
| *Tylosurus acus melanotus* | x,  x | x | x |
| *Ablennes hians* | 20.84,  x | MAB II: Original drawing by Smith NS. | x |
| *Fundulus diaphanus* | 10.19,  5.36 | www.fishbabies.ca/frwspecies.html | fishbase: Fdiaphanusmale.jpg by Terceira AC. |
| *Lucania parva* | 10.15,  x | MAB II: Foster N. 1974 | x |
| *Atherina presbyter* | 16.50,  6.51 | Palmer CJ, Culley MB. 1984 The egg and early life stages of the sandsmelt, Atherina presbyter Cuvier. *J Fish Biol*. 24(5), 537-544. | fishbase: Atpre_u0.jpg by Ostergaard T. |
| *Membras martinica* | x,  6.61 | x | http://txmarspecies.tamug.edu/ |
| *Dactylopterus volitans* | x,  7.11 | x | fishbase: Davol_m0.jpg by Freitas R. |
| *Hemitripterus americanus* | x,  5.20 | x | World Register of Marine Species: Fisheries and Oceans Canada, Wiele H. |
| *Eurypegasus draconis* | x,  6.67 | x | fishbase: Eudra_u5.jpg by Randall JE. |
| *Gasterosteus aculeatus* | 18.69,  6.52 | MAB II: Swarup H. 1958 | MAB II: Hildebrand SF, Schroeder WC. 1928 |
| *Sphoeroides maculatus* | 7.83,  6.79 | www.jeffbloom.net by Jeffrey Bloom 2010 | MAB VI: Welsh WW, Breder CM Jr. 1922 |
| *Balistes capriscus* | 3.46,  2.25 | Lyczkowski-Shultz J, Ingram GW. 2003 *Preliminary guide to the identification of the early life stages of balistid fishes of the western Central North Atlantic*. US Department of Commerce, NOAA, NMFS, Southeast Fisheries Science Center, Mississippi Laboratory. | fishbase: Bacar_u6.jpg by Cambraia Duarte, PMN. |
| *Chaenophryne longiceps* | x,  2.77 | x | fishbase: Chlon_u1.jpg by Dolgov A. |
| *Antennarius pauciradiatus* | x,  x | x | x |
| *Histrio histrio* | x,  2.56 | x | MAB VI: Jordan DS. 1905 |
| *Trichiurus lepturus* | x,  x | x | x |
| *Lepidopus caudatus* | x,  x | x | x |
| *Lepidocybium flavobrunneum* | x,  x | x | x |
| *Tetragonurus atlanticus* | x,  7.24 | x | MAB VI: Jordan DS, Evermann BW. 1896-1900 |
| *Peprilus paru* | x,  1.78 | x | MAB VI: Hilebrand S, Schroeder WC. 1928 |
| *Scomber scombrus* | x,  x | x | x |
| *Euthynnus affinis* | x,  x | x | x |
| *Lepomis cyanellus* | x,  3.00 | x | MAB III: Trautman MB. 1957 |
| *Epinephelus niveatus* | x,  3.88 | x | MAB II: Smith CL. 1971 |
| *Sphyraena borealis* | x,  8.37 | x | MAB VI: Goode GB. 1884 |
| *Sphyraena tome* | x,  x | x | X |
| *Mugil curema* | x,  5.37 | x | MAB VI: Goode GB. 1884 |
| *Perca flavescens* | 13.78,  4.81 | MAB III: Fish MP. 1929 | MAB III: Goode GB, etal. 1884 |
| *Lobotes surinamensis* | x,  2.81 | x | MAB III: Goode GB, etal. 1884 |
| *Pomacanthus rhomboides* | x,  1.62 | x | fishbase: Porhu_u1.jpg by Randall JE. |
| *Ocyurus chrysurus* | x,  4.09 | x | MAB III: Jordan DS, Evermann BW. 1896-1900 |
| *Lutjanus griseus* | x,  x | x | x |
